# Recent Advances in Bunyavirus Reverse Genetics Research: Systems Development, Applications, and Future Perspectives

**DOI:** 10.3389/fmicb.2021.771934

**Published:** 2021-12-07

**Authors:** Fuli Ren, Shu Shen, Qiongya Wang, Gang Wei, Chaolin Huang, Hualin Wang, Yun-Jia Ning, Ding-Yu Zhang, Fei Deng

**Affiliations:** ^1^Research Center for Translational Medicine, Wuhan Jinyintan Hospital, Wuhan, China; ^2^State Key Laboratory of Virology, Wuhan Institute of Virology, Chinese Academy of Sciences, Wuhan, China; ^3^National Virus Resource Center, Wuhan Institute of Virology, Chinese Academy of Sciences, Wuhan, China

**Keywords:** bunyavirus, reverse genetics, minigenome, iVLP, infectious full-length clone system

## Abstract

Bunyaviruses are members of the *Bunyavirales* order, which is the largest group of RNA viruses, comprising 12 families, including a large group of emerging and re-emerging viruses. These viruses can infect a wide variety of species worldwide, such as arthropods, protozoans, plants, animals, and humans, and pose substantial threats to the public. In view of the fact that a better understanding of the life cycle of a highly pathogenic virus is often a precondition for developing vaccines and antivirals, it is urgent to develop powerful tools to unravel the molecular basis of the pathogenesis. However, biosafety level −3 or even −4 containment laboratory is considered as a necessary condition for working with a number of bunyaviruses, which has hampered various studies. Reverse genetics systems, including minigenome (MG), infectious virus-like particle (iVLP), and infectious full-length clone (IFLC) systems, are capable of recapitulating some or all steps of the viral replication cycle; among these, the MG and iVLP systems have been very convenient and effective tools, allowing researchers to manipulate the genome segments of pathogenic viruses at lower biocontainment to investigate the viral genome transcription, replication, virus entry, and budding. The IFLC system is generally developed based on the MG or iVLP systems, which have facilitated the generation of recombinant infectious viruses. The MG, iVLP, and IFLC systems have been successfully developed for some important bunyaviruses and have been widely employed as powerful tools to investigate the viral replication cycle, virus–host interactions, virus pathogenesis, and virus evolutionary process. The majority of bunyaviruses is generally enveloped negative-strand RNA viruses with two to six genome segments, of which the viruses with bipartite and tripartite genome segments have mostly been characterized. This review aimed to summarize current knowledge on reverse genetic studies of representative bunyaviruses causing severe diseases in humans and animals, which will contribute to the better understanding of the bunyavirus replication cycle and provide some hints for developing designed antivirals.

## Introduction

As the handling of many infectious and highly pathogenic members of the order Bunyavirales is restricted to occurring in a laboratory with a biosafety level −3, or even −4, many studies focused on different aspects of the viral life cycle and pathogenesis have been hampered. Reverse genetics systems established up to now, such as the minigenome (MG), infectious virus-like particles (iVLP), and infectious full-length clone (IFLC) systems, have been efficient tools for researchers to conduct some important experiments at lower biocontainment than at level 3 or 4. Among these experimental systems constructed for bunyaviruses, MG and iVLP systems are used to model their partial life cycle, which enables the dissection of virus invasion (a process the virus enter the host cells), viral genome replication, transcription, ribonucleoprotein assembly, virion packaging, and budding processes. The IFLC system is used to rescue recombinant viruses, which has made it possible to manipulate viral genomes at the RNA/DNA molecular level. These established reverse genetics systems have exerted great potential to help us to better understand the bunyaviruses and to develop antivirals and vaccines.

Over the past few decades, reverse genetics technology has revolutionized the field of RNA virology, which has made it possible to manipulate RNA viral genomes and rescue genome analogs (referring to minigenome RNA in generally), infectious iVLP, and recombinant viruses from cDNA clones. Here, we summarize the already established MG, iVLP, and IFLC systems for bunyaviruses causing severe diseases in humans and animals. These bunyaviruses are mostly distributed in the families *Arenaviridae*, *Hantaviridae*, *Nairoviridae*, *Peribunyaviridae*, and *Phenuiviridae* (as shown in Table 1). Among members of these families, the viral genome of viruses belonging to *Hantaviridae*, *Nairoviridae*, *Peribunyaviridae*, and *Phenuiviridae* generally consists of three genome segments denoted as large (L), medium (M), and small (S), while the viral genome of viruses in *Arenaviridae* is mainly composed of two segments, i.e., L and S segments. These established systems suggest that the reverse genetics technology has been widely developed for bunyaviruses and widely used by researchers to dissect all aspects of the viral life cycle.

**Table 1:** Summary of the established reverse genetics systems for *Bunyavirales*.

**Virus**	**Genus**	**Family**	**Endemic area**	**Established systems**	**References**
Lassa virus (LASV)	*Mammarenavirus*	*Arenaviridae*	Western Africa	T7- and pol-I-driven MG, IFLC systems	Cai et al., 2018, 2020a
Lymphocytic choriomeningitis Mammarenavirus (LCMV)			Americas, Africa, Asia, and Europe	T7-driven MG, IFLC systems	Lee et al., 2000; Cheng et al., 2015
Tacaribe virus (TCRV)			America	pol-I-driven MG, IFLC systems	Ye et al., 2020
Lujo virus (LJV)			South Africa	T7-driven MG, IFLC systems	Bergeron et al., 2012
Junin virus (JUNV)			Argentina	pol-I-driven MG, IFLC systems	Emonet et al., 2011
Pichinde virus (PICV)			Guinea	T7-driven MG, IFLC system	Lan et al., 2009
Machupo virus (MACV)			South America	pol-I-driven MG, IFLC systems	Patterson et al., 2014
Bunyamwera virus (BUNV)	*Orthobunyavirus*	*Peribunyaviridae*	Africa	T7- and pol-I-driven MG, iVLP and IFLC systems	Weber et al., 2001; Shi et al., 2006; Mazel-Sanchez and Elliott, 2012
Shuni virus (SHUV)			South Africa	T7-driven IFLC system	Oymans et al., 2020
Akabane virus (AKAV)			Africa, Australia, and Asia	T7- and pol-I-driven IFLC system	Takenaka-Uema et al., 2015, 2016
LaCrosse virus (LACV)			North America	T7-driven MG, iVLP and IFLC systems	Blakqori and Weber, 2005; Klemm et al., 2013
Schmallenberg virus (SBV)			Europe	T7-driven IFLC system	Elliott et al., 2013; Varela et al., 2013
Cache Valley virus (CVV)			America	T7-driven MG and IFLC system	Dunlop et al., 2018
Kairi virus (KRIV)			America	T7-driven MG and IFLC systems	Dunlop et al., 2018
Oropouche virus (OROV)			Central and South America	T7-driven IFLC system	Tilston-Lunel et al., 2015
Hantaan virus (HTNV)	*Orthohantavirus*	*Hantaviridae*	Asia, Europe, and America	T7- and pol-I-driven MG system	Flick et al., 2003; Zhang et al., 2008
Andes virus (ANDV)			South America	T7-driven MG system	Brown et al., 2012
SFTS bandavirus (SFTSV)	*Bandavirus*	*Phenuiviridae*	East Asia	T7- and pol-I-driven MG, iVLP and IFLC systems	Brennan et al., 2015, 2017;Rezelj et al., 2019; Ren et al., 2020
Heartland virus (HRTV)			America	T7- and pol-I-driven MG, iVLP system	Rezelj et al., 2019; Ren et al., 2020
Uukuniemi Virus (UUKV)	*Phlebovirus*	*Phenuiviridae*	Europe, Asia	T7 and pol-I-driven MG, iVLP and IFLC systems	Flick and Pettersson, 2001; Flick et al., 2004; Overby et al., 2006; Rezelj et al., 2015
Rift Valley fever virus (RVFV)			Africa	T7- and pol-I-driven MG, iVLP and IFLC systems	Ikegami et al., 2006; Habjan et al., 2008, 2009; Smith et al., 2018; Jerome et al., 2019
Arumowot virus (AMTV)			Africa	T7-driven IFLC system	Hallam et al., 2019
Crimean-Congo hemorrhagic fever virus (CCHFV)	*Orthonairovirus*	*Nairoviridae*	Asia, Europe and Africa	T7-driven MG, iVLP and IFLC systems	Bergeron et al., 2015; Welch et al., 2020
Hazara virus (HAZV)			Pakistan	T7-driven MG and IFLC system	Fuller et al., 2019; Matsumoto et al., 2019
Tomato spotted wilt virus (TSWV)	*Orthotospovirus*	*Tospoviridae*	Australia, India and America	CUP1promoter-driven MG system	Ishibashi et al., 2017

This review provides an overview of the current status of reverse genetic studies on important bi- and trisegmented emerging and re-emerging bunyaviruses, recent advances in the applications of these systems. Furthermore, we also discuss the limitations of the currently established systems for bunyaviruses and unsolved problems that urgently need to be addressed.

## Classification And Genome Organization Of *Bunyavirales*

*Bunyavirales* is a newly proposed order consisting of related viruses distributed among 12 families: *Arenaviridae*, *Cruliviridae*, *Filoviridae*, *Hantaviridae*, *Leishbuviridae*, *Mypoviridae*, *Nairoviridae*, *Peribunyaviridae*, *Phasmaviridae*, *Phenuiviridae*, *Tospoviridae*, and *Wupedeviridae* proposed by the International Committee on Taxonomy of Viruses, of which the viral genome is composed of linear, segmented, single-stranded, negative-sense, or ambisense RNA genomes; this order is now comprised of 48 genera and over 383 species (Abudurexiti et al., 2019; Kuhn et al., 2020). Among the 12 families in the *Bunyavirales* order, *Arenaviridae*, *Hantaviridae*, *Nairoviridae*, *Peribunyaviridae*, and *Phenuiviridae* contain lots of important pathogens that are able to cause severe diseases in humans and animals with a worldwide geographical distribution.

Viruses from the family *Arenaviridae*, *Hantaviridae*, *Nairoviridae*, *Peribunyaviridae*, and *Phenuiviridae* are comprised of a variety of widely known representative pathogenic viruses, such as the Lassa virus (LASV, *Arenaviridae* family), which is endemic across large regions within Western Africa and is estimated to infect hundreds of thousands of individuals annually (Cai et al., 2020b). Hantaan virus (HTNV, *Hantaviridae* family) is found predominantly in Europe and Asia (Brocato and Hooper, 2019). Crimean-Congo hemorrhagic fever virus (CCHFV, *Nairoviridae* family) can infect human host primarily through infected ticks or by contact with infected hosts or their body fluids and tissues (Serretiello et al., 2020). Bunyamwera virus (BUNV, *Peribunyaviridae* family), which causes disease in livestock animals, avian species, and humans, is considered the prototype of the bunyavirus as it is the most characterized to date (Dutuze et al., 2018). Rift Valley fever virus (RVFV, *Phenuiviridae* family), which is found in Sub-Saharan Africa, is associated with disease in camels, cattle, goats, and sheep but also causes severe morbidity and mortality in humans (Paweska, 2015; Noronha and Wilson, 2017).

**Figure 1:**
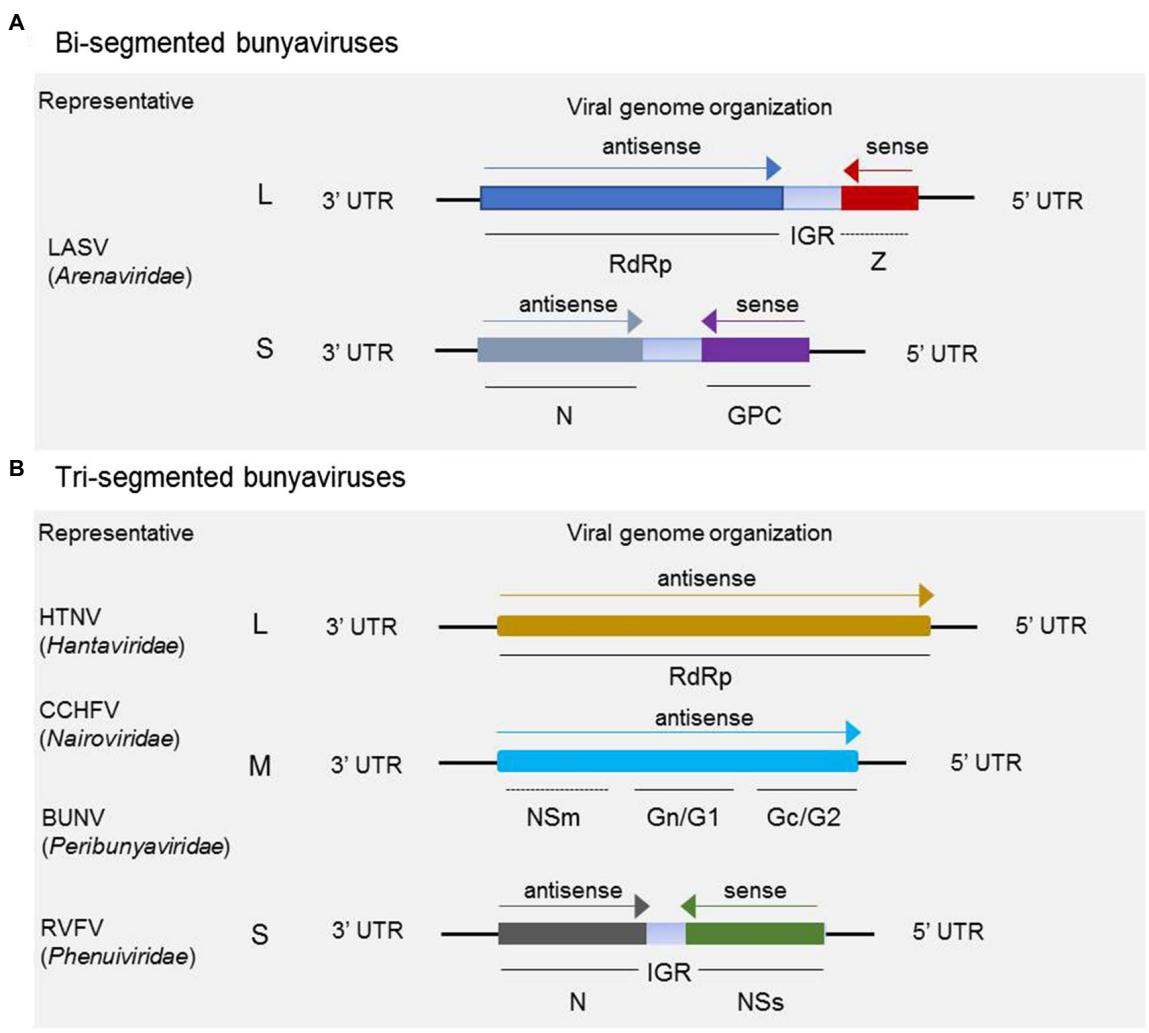
Schematic of genome organization and coding strategies of bisegmented and trisegmented bunyaviruses. For bisegmented bunyaviruses, in Lassa arenavirus (LASV), for example, the L and S are generally ambisense segments, of which the L codes for RdRp and a zinc-binding protein (Z), and S codes for glycoprotein precursor (GP) and nucleoprotein (N). The Z protein is only encoded by some members in *Mammarenavirus* and *Reptarenavirus* **(A)**; **(B)** for trisegmented bunyaviruses, such as Hantaan virus (HTNV), Crimean-Congo hemorrhagic fever virus (CCHFV), Bunyamwera virus (BUNV), and Rift Valley fever virus (RVFV), the viral genome consists of L, M, and S, of which the L codes for RdRp and M mainly codes for glycoprotein Gn (or G1) and Gc (or G2). For CCHFV, BUNV, RVFV, and several other members not shown here, the M segment also encodes NSm besides the glycoproteins. It should be noted that the NSm encoding gene of CCHFV and BUNV is both located between the encoding genes of Gn and Gc, which is different from the schematic shown for RVFV. The S segment adopts an ambisense coding strategy to encode nonstructural protein NSs and nucleoprotein N.

Bunyaviruses are mostly spherical virion with a lipid bilayer envelope containing segmented negative-sense or ambisense single-stranded RNA. For the majority in the five families discussed, the virus genomes comprise tripartite segments, except for some members in *Arenaviridae*, whose genomes consist of bipartite segments. For the majority of arenaviruses, the genome is generally comprised of bipartite segments denoted as L and S, of which the S encodes glycoprotein precursor (GP) and the nucleocapsid protein (N) in an ambisense arrangement, and L codes for the RNA-dependent RNA polymerase (RdRp) and a zinc-binding protein (Z) for some members in the genera *Mammarenavirus* and *Reptarenavirus* (as shown in Figure 1A). It has been demonstrated that the matrix Z-protein of virus from the genus *Mammarenavirus* species participates in the formation of infectious viral particles and viral budding during the life cycle (Perez et al., 2003; Pontremoli et al., 2019). As shown in Figure 1B, a typical three-segmented bunyavirus contains tripartite genome segments, and the genomic RNA segments are named according to their relative sizes, which are termed L, M, and S. L and S code for, respectively, RdRp and nucleoprotein (N), which participates in genome replication and transcription. S generally also encodes the nonstructural protein NSs, which participates in virus immune escape. M codes for a single-polyprotein precursor (GP), from which two surface GPs, such as Gn (or G2) and Gc (or G1), are produced by host cell proteases (Guardado-Calvo and Rey, 2017). In a few cases, an additional nonstructural protein NSm is also encoded by M. Some members of the genus *Orthobunyavirus* (BUNV for instance) and *Phlebovirus* (RVFV for instance) encode the NSm protein as part of a precursor with the glycoproteins, whereas NSm of virus from the genus *Orthotospovirus* (tomato spotted wilt virus for instance) is translated from a subgenomic messenger RNA (mRNA) in an ambisense manner (Guu et al., 2012).

## Viral Replication Cycle and Reverse Genetics Systems of Bunyaviruses

**Figure 2:**
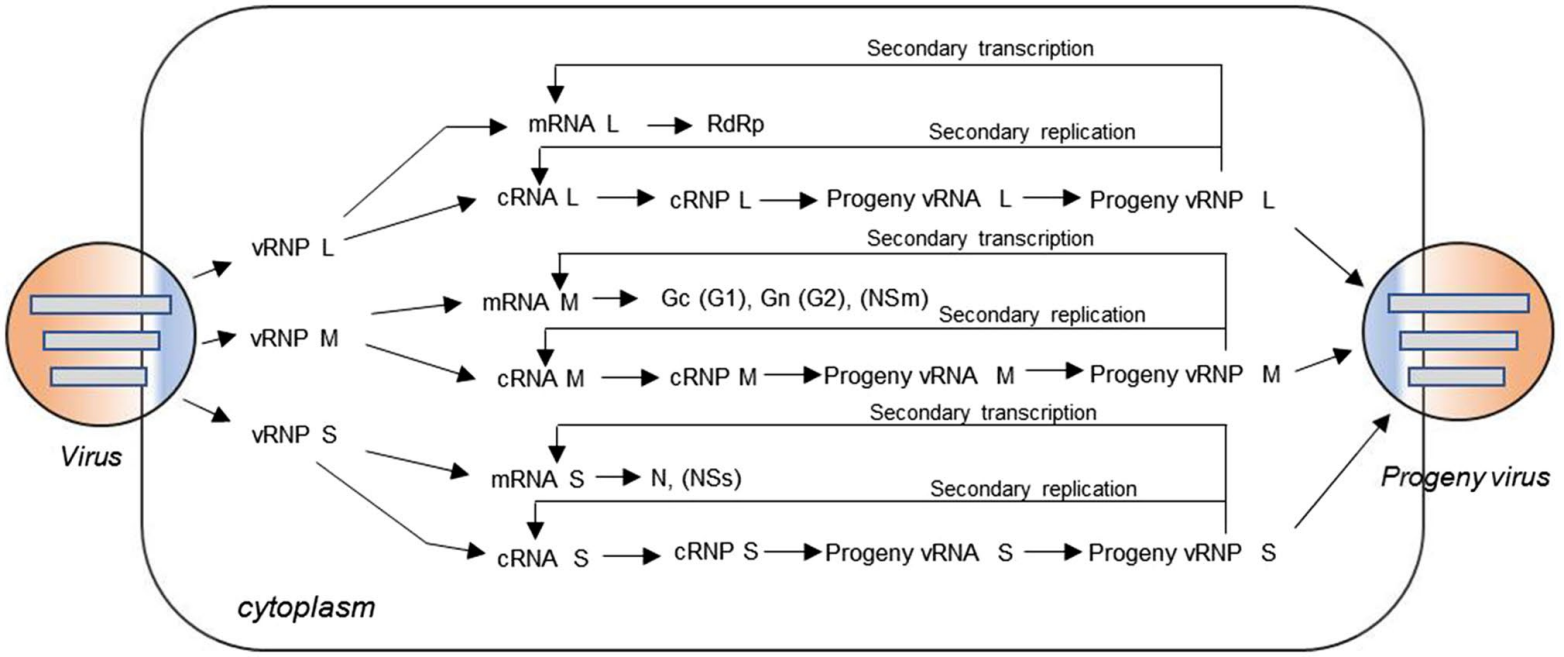
Virus replication cycle of typical three-segmented bunyaviruses in the cytoplasm. The viral genomic RNA is always encapsidated by N protein and associated with L protein to form ribonucleoprotein (RNP) complexes in the virion. After virus entry, uncoating occurs and the genomic RNAs with some specific viral proteins can be used to initiate transcription of positive-sense messenger RNA (mRNA) and the intermediate product cRNA. The mRNA can serve as a template for viral protein synthesis, such as L protein, Gc (or G1), Gn (or G2), NSm, N, and NSs; and the cRNA coated by N can recruit L protein to form functional cRNPs, which can serve as templates to synthesize progeny vRNAs. Similarly, after recruitment and encapsidation, the progeny vRNPs are formed and then packaged by glycoprotein into progeny viruses. Meanwhile, the nascent vRNP can also be used as templates to synthesize cRNA and mRNA through the so-called secondary replication and secondary transcription.

Genomes of bunyaviruses are comprised of linear single-stranded negative-sense RNA, and the viral genomic RNA segments always contain untranslated regions (UTRs) on both the 5′ and 3′ ends that are denoted as 5′ UTR and 3′ UTR, which play important roles in the virus life cycle by promoting transcription, replication, and encapsidation of the viral genome. Bunyaviruses replicate exclusively in the host cell cytoplasm, with maturation and budding occurring in the Golgi apparatus, and are released *via* cellular export pathways (Hoenen et al., 2011). The viral genomic and antigenomic RNAs are always encapsidated by N and associated with RdRp to form ribonucleoprotein (RNP) complexes both within the host cell and in the virion. These RNP particles are able to serve as functional templates to synthesize positive-sense mRNA and progeny virus genome RNA with the assist of the viral polymerase (Sun et al., 2018). The bunyaviruses enter a host cell through a glycoprotein-mediated virus binding process. Once this process is finished, the viral genomic RNAs will undergo an uncoating process to initiate the transcription to generate mRNA and the intermediate products cRNA with the assist of some specific viral proteins (Guu et al., 2012; Ferron et al., 2017; Sun et al., 2018). Initiation of transcription of the viral mRNAs is primed by short sequences derived from the 5′ end of host mRNAs through a so-called cap-snatching mechanism (Simons and Pettersson, 1991). The mRNA can serve as a template for viral protein synthesis, such as RdRp, Gn (or G2), Gc (or G1), NSm, N, and NSs; and the cRNA coated by N can recruit RdRp to form functional cRNPs, which can serve as templates to synthesize progeny vRNAs. Similarly, after the recruitment and encapsidation, the progeny vRNPs are formed and can be packaged by glycoprotein into progeny virus. Meanwhile, the progeny vRNPs can also be used as templates to synthesize mRNA and cRNA *via* so-called secondary transcription and secondary replication. The replication cycle for a typical trisegmented bunyavirus is shown in Figure 2, and there are very few differences for a bisegmented bunyavirus.

According to the viral replication cycle and the initial transcripts of the cDNA clones, two categories of reverse genetics systems can be constructed for negative-strand RNA bunyaviruses, including sense (−) and antisense (+) MG, iVLP, and IFLC systems, of which the initial transcripts are genomic RNA (vRNA) and antigenomic RNA (cRNA), respectively. It is theoretically possible that both (+) and (−) systems can successfully model the partial or entire virus replication cycle. It has been demonstrated that the sense (+) and antisense (−) reverse genetics systems of Uukuniemi virus (UUKV) show no obviously different efficiencies in rescuing MG RNAs and the recombinant virus (Flick and Pettersson, 2001; Flick et al., 2004; Overby et al., 2006; Rezelj et al., 2015).

## Current Status of Reverse Genetics Research on Bunyaviruses

Currently, the definition of reverse genetics is not just used exclusively to describe the generation or rescue of replication competent viruses from cDNA clones; a broader definition consisting of the generation and subsequent replication and transcription of full-length virus RNA genomes or truncated genome analogs from cDNA clones has been widely recognized by researchers (Hoenen et al., 2011). According to the broader definition, the reverse genetics systems constructed for bunyaviruses mainly consist of MG (or minireplicons), iVLP [or transcription and replication competent virus-like particles (trVLPs)], and IFLC systems. The reverse genetic systems developed for segmented bunyaviruses mainly rely on the production of viral transcripts from transfected plasmids by either bacteriophage T7 RNA polymerase or cellular RNA polymerase I (pol-I; Walpita and Flick, 2005). In a T7 RNA polymerase-driven reverse genetics system, the viral transcripts are produced in the cytoplasm, whereas RNA pol-I is a cellular protein that synthesizes unmodified RNA species with defined terminal sequences in the nuclei of transfected cells. Moreover, it has been reported that the 5′-triphosphate group of T7 pol transcripts strongly activates the antiviral interferon system *via* the intracellular RNA receptor RIG-I (Hornung et al., 2006). However, it has been demonstrated that the T7- and pol-I-driven reverse genetics systems are equally efficient in rescuing MG RNAs and recombinant virus for trisegmented RVFV (Habjan et al., 2008) and bisegmented lymphocytic choriomeningitis mammarenavirus (LCMV; Flatz et al., 2006; Sanchez and de la Torre, 2006).

**Figure 3:**
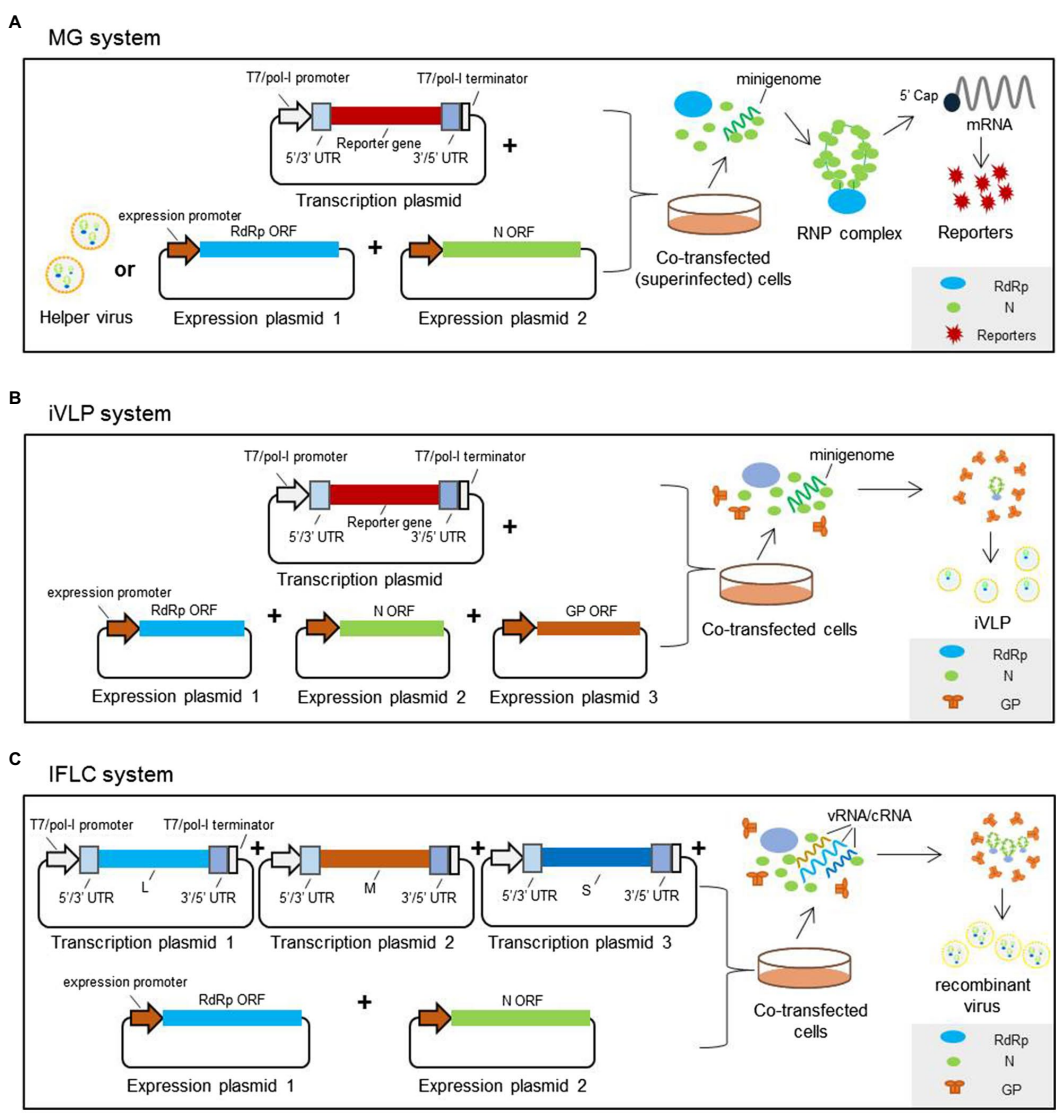
Reverse genetics systems established for typical three-segmented bunyaviruses. **(A)** Minigenome system based on plasmid transfection mainly consisting of expression plasmids encoding RdRp, N, and T7 or pol-I-driven transcription plasmid to express virus-like genome or antigenome RNA. The transcription plasmids are constructed by inserting the reporter-encoding gene flanked by viral UTRs into a T7 or pol-I promoter-driven vector in sense or antisense orientation. The expression plasmids are constructed by inserting the RdRp- or N-encoding gene into an expression vector. While in a minigenome system with helper virus, the RdRp and N are provided by the authentic wild-type virus instead of the expression plasmids. After co-transfection of the plasmid mix, the functional RNP complexes can form, leading to the expression of reporter proteins. **(B)** The infectious virus-like particle system is constructed based on the minigenome system, in which Gn/Gc proteins are also provided in trans form from expression plasmids to package the functional RNP complexes to form iVLPs. **(C)** In an IFLC system, after the cells are co-transfected with plasmids to express full-length antigenomic or genomic L, M, and S and plasmids to express RdRp and N, the functional L-, M-, and S-RNP complexes form. The RNP particles are then packaged by glycoproteins to generate infectious recombinant viruses.

For a typical three-segmented bunyavirus, schematic organization of reverse genetics systems, including MG, iVLP, and IFLC systems, can be seen in Figure 3. Briefly, RdRp, N, and Gn/Gc proteins are provided in trans form from Pol-II or T7-driven expression plasmids or from the helper virus and the genomic or antigenomic RNA segments, which are generated from a T7 or pol-I-driven expression plasmid. The MG system is mainly comprised of two expression plasmids to encode RdRp and N, and one transcription plasmid to express virus-like genome or antigenome RNA (as shown in Figure 3A). For the iVLP system, the expression plasmid encoding glycoprotein Gn/Gc is added to generate iVLPs based on the MG system (as shown in Figure 3B). For the IFLC system, expression plasmids encoding viral RdRp, NP, and sometimes Gn/Gc are also included, and transcription plasmids to express viral genomic or antigenomic L, M, and S are co-transfected into targeted cells to generate infectious recombinant viruses (as shown in Figure 3C). However, in BSR-T7/5 cells, the recombinant BUNV can be generated by transfecting just three antigenomic plasmids under the control of the T7 promoter, though these antigenomic plasmids provide low levels of supporting proteins (Lowen et al., 2004).

The segmented nature of the bunyavirus genome potentially contributes to easy reassortment between two genetically related viruses when they co-infect the same host cell (Briese et al., 2013). To evaluate the genome reassortment potential and model the evolutionary process between closely related bunyaviruses, the so-called combinatory MG and iVLP systems based on traditional MG and iVLP systems have been developed for several important bunyaviruses, such as severe fever with thrombocytopenia syndrome virus (SFTSV), Heartland virus (HRTV; Rezelj et al., 2019; Ren et al., 2020), Oropouche virus (OROV), Schmallenberg virus (SBV; Tilston-Lunel et al., 2017), Cache Valley virus (CVV), and Kairi virus (KRIV; Dunlop et al., 2018). The combinatorial MG or iVLP system is generally comprised of the RdRp and N from the same virus (virus A for instance), the vRNA/cRNA-MG from a closely related virus (virus B for instance), and the glycoprotein (omitted from the MG system) from virus A or B. Results of our previous studies have revealed that the RdRp and N of SFTSV and HRTV cannot cross-recognize each other in a murine pol-I-driven MG system though the NP of SFTSV and another novel *Banyangvirus* Guertu virus (GTV) can efficiently substitute for each other to form functional RNP complex (Shen et al., 2018). However, Dunn et al. have reported that the BUNV RdRp can also transcribe the MG-RNA in concert with the N proteins of closely related bunyaviruses, such as Batai virus, CVV, Maguari virus, Main Drain virus, and Northway virus, which prove that bunyavirus RdRp sometimes can also interact with the N from another virus.

## Development and Application of Reverse Genetic Systems For Bunyaviruses

### Minigenome Reporter Systems

MGs or minireplicons are virus-like genome segments (or genome analogs) that contain the *cis*-acting elements required for replication and transcription, but in which some or all of the viral protein-coding regions have been replaced with genes encoding reporter proteins, such as enhanced green fluorescent protein (*EGFP*), chloramphenicol acetyltransferase, or luciferase. Viral proteins, such as RdRp and N, required for the replication and transcription of MG, are provided in trans form to drive expression of the encoded reporter protein, and viral proteins can be provided *via* either a co-infecting helper virus or by expression plasmids. MG systems are generally used as model systems for virus genome transcription and replication, and are frequently, although not always, generated as precursors to the development of IFLC systems.

It is incontestable that a breakthrough came when Palese et al. established the first MG system for the modification of a negative-sense segmented RNA virus, influenza A virus, in 1989 (Luytjes et al., 1989). However, the reliance on helper virus infection restricted its applications. For negative-sense RNA viruses, the first established plasmid-based MG system was pioneered by Pattnaik and Wertz et al., who established a replication system to study the structure–function relationships between viral proteins and RNA replication (Pattnaik and Wertz, 1990, 1991). For bunyaviruses, the first established MG system based on the T7 promoter and BUNV S segment was established by Dunn et al. (1995); thereafter, MG systems have been advancing rapidly and widely applied by researchers to investigate the transcription, replication, and pathogenesis of bunyaviruses.

The MG system has been mainly used to examine the *cis*- and *trans*-acting factors involved in bunyavirus replication and transcription (Barr et al., 2003; Ikegami et al., 2005), defining the minimal *cis*- and *trans*-acting factors required for viral replication (Barr et al., 2003), mapping critical residues of the viral promoter (Flick et al., 2004; Zhao et al., 2013), exploring the packaging (Kohl et al., 2006), revealing the pathogenic mechanism (Ruiz-Jarabo et al., 2003; Shao et al., 2018), and identifying novel antiviral compounds (Rathbun et al., 2015) without requiring the use of live forms of bunyaviruses. For example, Flick et al. created the first pol-I-driven MG system of HTNV (Hantaviridae) in 2003, which provided powerful tools for studying the functions of essential genes or proteins of hantavirus (Flick et al., 2003). The first pol-I promoter-based cRNA and vRNA MG systems of CCHFV (family Nairoviridae) were constructed with a helper virus in 2003, and this is also the first reported reverse genetics system for nairoviruses. Then, Bergeron et al. developed a more efficient CCHFV helper virus-independent MG system based on S, M, and L segments for the analysis of virus RNA and protein features involved in CCHFV replication. Using these systems, they revealed that UTRs of CCHFV L, M, and S were similar in support of replication of the respective MGs, and the ovarian tumor protease activity from the L protein was dispensable for virus RNA replication (Bergeron et al., 2010). The first reported MG system of the prototype bunyavirus BUNV (*Peribunyaviridae*) was pioneered by Dunn et al. in 1995, and they performed a wide-scale mutagenic analysis of viral N to examine the effects of different N mutants on viral RNA transcription and replication using the T7-driven MG system (Dunn et al., 1995). Over the next several years, T7-driven MG systems have been developed for many other viruses from the genus *Peribunyaviridae*, such as Shuni virus (SHUV), AKAV, LACV, SBV, CVV, KRIV, and OROV (as shown in Table 1), and these systems have been widely used to study the viral replication cycle. For viruses from the genus *Phenuiviridae*, the first successfully constructed MG system was reported in 1995 for RVFV based on the classical T7-vaccinia virus (T7-VV) system, where it was proven that viral N and L are absolutely required and sufficient to reconstitute the transcriptase activity (Lopez et al., 1995). Thereafter, T7- and/or pol-I-driven MG systems have been successively developed for UUKV, SFTSV, and HRTV (see Table 1).

### Infectious Virus-Like Particle System

MG system has been used to model the replication and transcription process of the viral life cycle, but it cannot mimic other steps, such as virus entry, budding, and packaging. Therefore, the iVLP or trVLP system has been developed to generate single-cycle infection particles. As this system can model a complete single infectious cycle of bunyavirus, it has become a very effective tool to further our understanding of every aspect of the virus life cycle.

Currently, a variety of iVLP systems have been developed for bunyaviruses, such as BUNV, RVFV, UUKV, CCHFV, and SFTSV, which belong to *Peribunyaviridae*, *Nairoviridae*, and *Phenuiviridae*, respectively. The first iVLP system was successfully constructed for BUNV (*Orthobunyavirus*) by Shi et al., (2006), which was used to investigate the role of the nonstructural protein NSm in virus assembly and morphogenesis (Shi et al., 2006). Using an iVLP assay, Feng et al. revealed that interferon-stimulated genes 20 (ISG20) can strongly inhibit gene expression from all three viral segments of BUNV in a recent study (Feng et al., 2018). An iVLP system for UUKV (*Phlebovirus*) was also constructed in 2006, and the authors reported that the RNP complexes are incorporated into iVLPs but are not required for the generation of particles. Morphological analysis of these particles by electron microscopy revealed that iVLPs, either with or without MGs, display a surface morphology indistinguishable from that of the authentic UUKV and that they bud into Golgi vesicles in the same way as UUKV does (Overby et al., 2006). In the case of RVFV (*Phlebovirus*), Habjan et al. developed an iVLP system and revealed that a human interferon-induced protein, MxA, inhibits both primary and secondary transcriptions of RVFV (Habjan et al., 2009). Based on the previous results, the authors further evaluated the potential of RVFV iVLPs being developed as a vaccine candidate and found that the RVFV iVLPs were highly immunogenic and conferred protection against RVFV infection in mice without exhibiting any side effects (Naslund et al., 2009). For CCHFV (*Orthonairovirus*), a so-called transcription and entry-competent virus-like particle (tc-VLP) system first developed in 2014, the authors revealed that the endonuclease domain of CCHFV L protein is located around amino acid D693 using this system (Devignot et al., 2015). Furthermore, the immunogenicity and protection were also investigated using the CCHFV tc-VLP as a vaccine candidate in an interferon alpha receptor knockout mouse model, and the results showed that mice vaccinated with a specific DNA vaccine combined with the tc-VLP were fully protected (Hinkula et al., 2017). In a recent study, the authors revealed the important role of CCHFV GP38 and NSm on CCHFV particle production and infectivity using the tc-VLP system (Freitas et al., 2020). An iVLP system of SFTSV (*Bandavirus*) based on the M segment was developed by Rezelj et al. in 2019 and revealed the reassortment potential of SFTSV and the closely related bandaviruses and phleboviruses (Rezelj et al., 2019).

Besides the classical reverse genetics systems, the so-called combinatorial MG and iVLP systems that contain viral components derived from closely related viruses were also established. Recently, Rezelj and Ren et al., respectively, developed T7- and pol-I-driven combinatorial MG and iVLP systems to study the *Bandavirus* (SFTSV, HRTV, and GTV) and *Phlebovirus* (RVFV and UUKV) genome reassortment potential (Rezelj et al., 2019; Ren et al., 2020). Similarly, other studies have also investigated the genome reassortment among orthobunyaviruses using combinatorial MG and iVLP systems, such as for OROV, SBV, and others within the Simbu serogroup (Tilston-Lunel et al., 2017). Since then, the reassortment potential of CVV and KRIV has also been evaluated (Dunlop et al., 2018). These combinatorial systems are excellent tools for researchers to assess the viral genome reassortment potential through evaluating the compatibility of viral components coming from closely related bunyaviruses without too much emphasis on the biosafety risk caused by co-infection. Meanwhile, they also have been used to identify the factors that affect the genome reassortment potential between different members of *Bunyavirales*.

### Infectious Full-Length Clone System

The IFLC system generally consists of expression plasmids encoding RdRp and N, and transcription plasmids based on cellular pol-I or T7 promoter expressing all viral genome segments; this system has always been used to generate infectious wild type or recombinant viruses and has tremendous potential in studying the virus replication cycle in its entirety. Today, IFLC systems for many members in the families of *Arenaviridae*, *Hantaviridae*, *Nairoviridae*, *Peribunyaviridae*, and *Phenuiviridae* have been successfully developed and widely applied to study various aspects of bunyaviruses.

Bridgen and Elliott made an important breakthrough in 1996 by successfully rescuing infectious BUNV entirely from cloned cDNAs under the control of T7 promoter. However, the procedure, including infection with a recombinant virus that expresses T7 RNA polymerase, transfection with three helper plasmids to express all of the BUNV proteins, and a second transfection with a mixture of plasmids to generate full-length antigenome viral RNAs, was cumbersome and the efficiency of generating infectious viruses was rather low (Bridgen and Elliott, 1996). With the IFLC system, they generated mutant BUNV without expressing NSs (nonstructural protein) and then revealed that although the NSs of BUNV are not essential for virus growth in tissue culture or mice, it has several functions in the virus life cycle and contributes to viral pathogenesis (Bridgen et al., 2001). As the complicated procedure and low rescue efficiency limit its application, Lowen et al. then improved the IFLC system of BUNV by replacing the recombinant virus with BSR-T7/5 cell lines to express T7 RNA polymerase. Then, the recombinant BUNV can be efficiently recovered following transfection with a mixture of plasmids, including two helper-protein (RdRp and N) expression plasmids and three ribozyme plasmids expressing L, M, and S antigenome RNAs. Interestingly, the BUNV can also be recovered following transfection with just three ribozyme plasmids without the need for separate helper-protein expression plasmids, which may be ascribed to the positive-sense transcripts generated by T7 RNA polymerase appearing to act as both messenger and antigenome RNA (Lowen et al., 2004). From then on, the five or three plasmids rescue system based on promoter T7 or pol-I has been widely used to generate wild type and recombinant bunyaviruses (see Table 1).

The IFLC system has been applied to facilitate the development of novel recombinant viruses for many pathogenic members of the families *Arenaviridae*, *Hantaviridae*, *Nairoviridae*, *Peribunyaviridae*, and *Phenuiviridae*, and the recombinant virus has been used to study the viral life cycle and develop vaccine candidates. A variety of approaches based on reverse genetics has been undertaken to use recombinant viruses to rapidly generate live-attenuated vaccine candidates and the strategies have been reviewed elsewhere (Tercero and Makino, 2020). Therefore, here, we just highlight the applications of IFLC systems by providing several representative examples. For the prototypic arenavirus LCMV, a live-attenuated vaccine candidate was developed in 2015 based on the presence of the noncoding intergenic region of the S genome segment in both L and S segments using IFLC systems (Iwasaki et al., 2015). The same strategy was recently utilized by others to develop a safe and effective recombinant virus vaccine candidate for LASV, which is a pathogenic arenavirus that causes Lassa fever with high morbidity and lethality, and there is no approved LASV vaccine currently available (Cai et al., 2020a). Based on the reverse genetics systems, Cai et al. also generated a recombinant LASV expressing a codon-deoptimized GP gene, which also has the potential to be developed as an excellent live-attenuated vaccine candidate (Cai et al., 2020b). For another high-pathogenic bunyavirus RVFV (genus *Phlebovirus*, family *Phenuiviridae*), the classical live-attenuated vaccine candidates, including recombinant virus with genome segment rearrangement (Wichgers Schreur et al., 2014) and single-cycle replicable virus (Murakami et al., 2014), were also successfully generated based on the IFLC system. These studies suggest that the IFLC systems can be used as excellent tools to rapidly develop vaccine candidates for emerging bunyaviruses, as long as a proper strategy is adopted.

Meanwhile, the rescued recombinant bunyaviruses with reporters can also be used as powerful tools to allow high-throughput screening for host factors affecting virus replication and antiviral drugs. Taking the prototype bunyavirus BUNV as an example, using a recombinant BUNV with expressing *EGFP*, the authors screened a library of more than 350 human ISGs for the effects on virus replication, and IFITM3 and IRF1 were identified as modest inhibitors of BUNV replication (Schoggins et al., 2014). Then, they increased the library size to 488 unique ISGs and found that the antiviral exonuclease ISG20 had broad-spectrum antiviral activity against multiple bunyaviruses, including BUNV, HRTV, SFTSV, and SBV (Feng et al., 2018). For most bunyaviruses, nonstructural proteins generally act as pathogenicity and virulence factors and a lot of IFLC systems have also been used to study the NSs or NSm of bunyaviruses, such as BUNV (Bridgen et al., 2001; Weber et al., 2002), UUKV (Rezelj et al., 2015), SFTSV (Brennan et al., 2017), RVFV (Bird et al., 2008, 2011), and SHUV (Oymans et al., 2020).

## Current Limitations and Future Perspectives

It is undisputed that the above studies on MG, iVLP, and IFLC systems highlight the importance of reverse genetics in improving our understanding of negative RNA viruses at the molecular level. Indeed, the established reverse genetics systems for bunyaviruses have been powerful tools for researchers to study bunyaviruses and develop antiviral countermeasures. However, in many situations, cautionary notes need to be introduced in interpreting the data obtained using reverse genetics approaches since there are many differences between modeling systems and the authentic viral replication cycle.

The reverse genetics systems based on plasmid transfection express the naked MG/viral RNA *via* T7 or cellular pol-I promoter and the naked RNAs must undergo artificial encapsidation by N that is also expressed by transfected plasmid. As there is no strictly equivalent process during virus infection, there may be many factors affecting the activity of reverse genetics systems rather than virus replication. For example, it has been reported previously that the NSs protein of both BUNV and RVFV decrease MG-encoded activity through inhibiting the viral polymerase (Weber et al., 2001; Brennan et al., 2011). In contrast, other studies have shown that expression of the RVFV NSs protein in a MG system enhances RNA replication and transcription, as measured by an increase in reporter gene activity (Ikegami et al., 2005). Interestingly, the NSs proteins of both BUNV and RVFV have been reported to be able to promote virus replication through counteracting the host antiviral responses during infection (Weber et al., 2002; Billecocq et al., 2004). These results suggest that conflicting data may be produced using different experimental systems, especially when the MG or iVLP system is used to conduct similar studies. When dealing with these problems, the different details between plasmid transfection-based reverse genetics systems and authentic virus infection should be taken into account. The IFLC systems based on transfection can be used to generate nascent wild type or recombinant viruses that can perform all the basic steps of the authentic viral replication cycle. It seems that there are very few limitations when dissecting the virus life cycle with the IFLC systems, as long as mutations introduced into the virus genome do not render the virus replicative incompetent. However, if the recombinant virus cannot be generated because of introduced mutations, it will be hard to determine which specific step of the viral replication cycle was interrupted without using the MG or iVLP system.

Although the reverse genetics systems have been successfully constructed for an increasing number of emerging bunyaviruses and have been used to dissect the viral life cycle, many questions remain to be answered in the future. For the MG and iVLP systems, it remains uncertain how the viral N selectively recognizes the foreign part of the MG RNAs despite there being huge differences between viral and reporter genes. It has been demonstrated that RdRp and N can recognize the heterologous MG RNAs to form functional RNP complexes and these combinatory RNPs can also be packaged by heterologous glycoprotein (Rezelj et al., 2019; Ren et al., 2020), of which the molecular mechanisms also wait to be revealed through utilizing reverse genetics systems. Moreover, in the case of bunyavirus packaging, it also remains to be elucidated whether the progeny virions package viral segments intricately in a selective manner or in a random packaging manner. Based on better understanding of the common characteristics of bunyaviruses, the bivalent or polyvalent vaccines against closely related emerging bunyaviruses may be rapidly generated in future, which will provide us with timely and effective protection when faced with an outbreak of a pathogenic bunyavirus.

## Conclusion

The advent of reverse genetics technology has greatly facilitated our understanding of the life cycle of negative viruses, including the largest group of RNA viruses, bunyaviruses. This review focused mainly on members of the families *Arenaviridae*, *Hantaviridae*, *Nairoviridae*, *Peribunyaviridae*, and *Phenuiviridae*, which are comprised of a large group of important pathogens infecting humans and animals and posing a threat to global health, food, and economy. The majority of the bunyaviruses is restricted to be handled in containment conditions, which has stunted the better understanding of the viral life cycle and the development of antivirals.

The reverse genetics system provides a powerful platform, allowing researchers to work with highly pathogenic viruses at a lower biocontainment level than normally required. Here, we not only summarized the already developed reverse genetics systems, including MG, iVLP, and IFLC, for important bunyaviruses but also provided a brief overview of the wide applications of these systems and future perspectives. These exciting research results can provide a foundation to fully exploit the potential of these reverse genetics systems and develop reverse genetics systems for other novel segmented negative-stranded RNA viruses.

## Author Contributions

FD, D-YZ, HW, Y-JN, CH, and FR conceived and designed the review and wrote the manuscript. QW and GW assisted with reference collection. FR and SS wrote and proofread the original manuscript. All authors contributed to the article and approved the submitted version.

## Funding

This work was funded by the National Major Scientific and Technological Special Project for “Significant New Drugs Development” (2020ZX09201-001), the National Natural Science Foundation of China (U20A20135), the National Program on Key Research Project of China (2018YFE0200402), the Innovation Team Project of Hubei Provincial Health Commission (WJ2019C003), the Special Project of Hubei Science and Technology Innovation Platform (2020DFE018), and the Biological Resources Programme, Chinese Academy of Sciences (KFJ-BRP-017-74).

## Conflict of Interest

The authors declare that the review was conducted in the absence of any commercial or financial relationships that could be construed as a potential conflict of interest.

## Publisher’s Note

All claims expressed in this article are solely those of the authors and do not necessarily represent those of their affiliated organizations, or those of the publisher, the editors and the reviewers. Any product that may be evaluated in this article, or claim that may be made by its manufacturer, is not guaranteed or endorsed by the publisher.
